# Multimodal Imaging in a Case of Chronic Sympathetic Ophthalmia

**DOI:** 10.7759/cureus.42645

**Published:** 2023-07-29

**Authors:** Abhijaat Chaturvedi, Shweta Parakh, Shrutanjoy Das, Vaibhav Bhatt, Gaurav Luthra, Saurabh Luthra

**Affiliations:** 1 Ophthalmology, Drishti Eye Institute, Dehradun, IND

**Keywords:** indocyanine green angiography (icga), fundus fluorescein angiography (ffa), systemic immunosuppressants, granulomatous panuveitis, chronic uveitis, sympathetic ophthalmia

## Abstract

We report a case of chronic sympathetic ophthalmia (SO) in a one-eyed patient who was successfully managed with systemic immunosuppression therapy. A 77-year-old one-eyed female presented with progressive diminution of vision in the left eye (OS) for one month. She had previously undergone a right eye (OD) pars plana vitrectomy elsewhere for exogenous post-operative endophthalmitis (after manual small incision cataract surgery five months ago), following which she developed phthisis. Granulomatous panuveitis and advanced cataract were noted in the OS. Findings on multimodal imaging, including spectral domain optical coherence tomography (SD-OCT), fundus fluorescein angiography (FFA), indocyanine green angiography (ICGA), and B-scan ultrasonography, were consistent with those of chronic SO. Promptly, oral steroids and systemic immunosuppressants were initiated under the supervision of a rheumatologist. At the three-week follow-up, complete resolution of clinical signs was observed on multimodal imaging. Chronic SO may present with ambiguous clinical signs, leading to a diagnostic dilemma. This may cause a delay in initiating treatment, which can prove to be highly detrimental, especially in one-eyed patients. Multimodal imaging is critical in excluding differential diagnoses and proves to be indispensable in the timely management of this sight-threatening condition.

## Introduction

Sympathetic ophthalmia (SO) is a rare, bilateral diffuse granulomatous panuveitis that generally occurs following penetrating ocular trauma or surgery involving the uvea in one eye. The eye that experiences the injury is referred to as the exciting eye, while the fellow eye may develop inflammation (days to years later) and is termed the sympathizing eye [1]. The diagnosis of this sight-threatening entity is based on clinical findings rather than serologic lab testing. We review the utility of multimodal imaging in the diagnosis and management of chronic SO in a one-eyed patient, treated successfully with immunosuppression therapy.

## Case presentation

A 77-year-old female presented with complaints of gradual, progressive diminution of vision in the left eye (OS) for one month. The right eye (OD) was phthisical. The patient had undergone manual small incision cataract surgery in OD at another location five months ago, following which she developed exogenous post-operative endophthalmitis. She subsequently underwent pars plana vitrectomy in OD elsewhere, but unfortunately, it led to the development of phthisis bulbi in the same eye.

At the time of presentation, the patient's best-corrected visual acuity (BCVA) was 3/60 OS. The intraocular pressure measured 16 mmHg in the OS. Slit lamp examination of the OS revealed diffuse mutton-fat keratic precipitates, 2+ anterior chamber cells, pigment on the anterior lens capsule, and advanced cataract with nuclear sclerosis grade 3-4. Indirect ophthalmoscopy of the OS revealed significant media haze due to cataract and a faintly visible hyperemic optic disc. Unfortunately, anterior segment imaging was not possible during this visit due to photophobia and patient discomfort.

**Figure 1:**
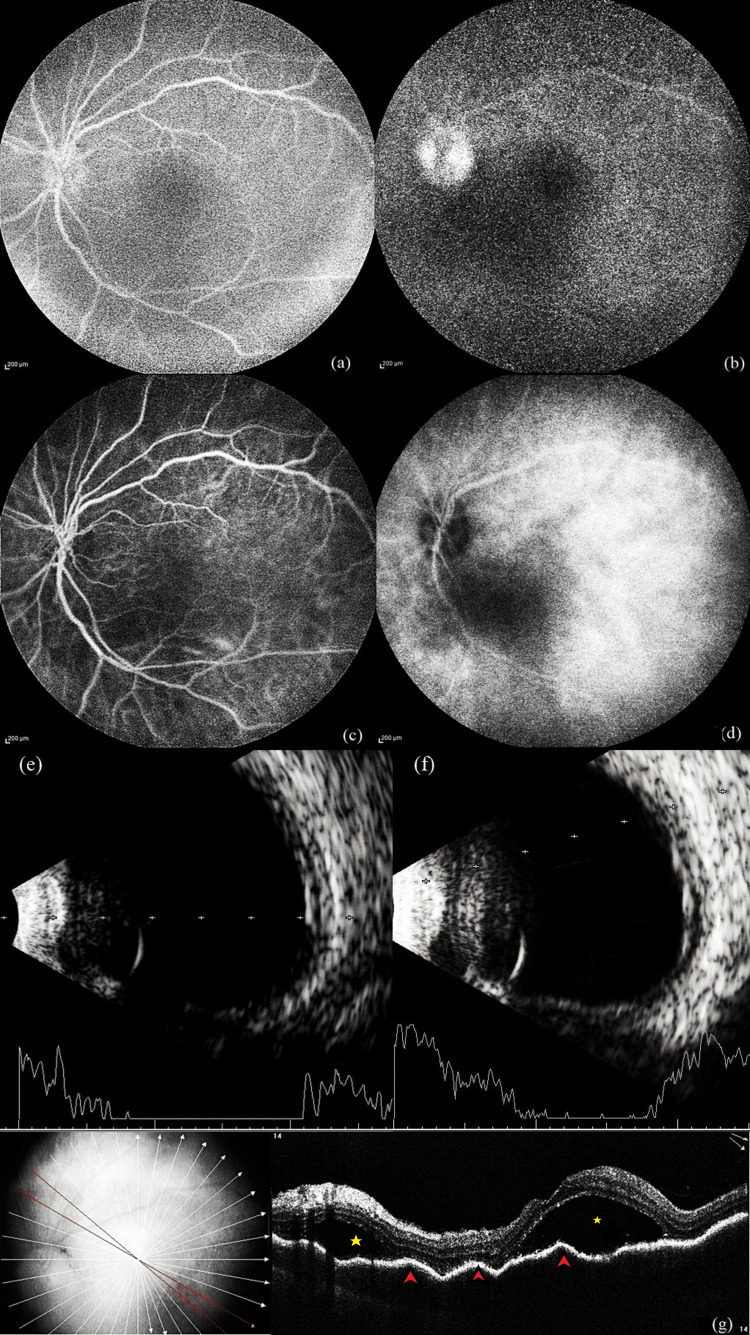
Multimodal imaging of left eye with chronic sympathetic ophthalmia at the first visit. (a, b) Fluorescein angiography showing diffuse hyperfluorescence at the posterior pole in both the early and late phases, with prominent disc staining in the late phase. (c, d) Indocyanine green angiography showing diffuse hypercyanescence in both the early and late phases. (e, f) B-Scan ultrasonography revealing diffuse retinochoroidal thickening with areas of exudative retinal detachment. (g) Spectral-domain optical coherence tomography displaying loss of the normal choroidal vascular pattern, retinal pigment epithelium undulations (indicated by red arrowheads), subretinal fluid pockets (denoted by yellow asterisks), and a fine epiretinal membrane.

Fundus fluorescein angiography (FFA) using Heidelberg Retina Angiograph (Heidelberg Engineering, Heidelberg, Germany) demonstrated diffuse early and late hyperfluorescence at the posterior pole, corresponding to areas of exudative retinal detachment, and prominent disc hyperfluorescence in the late phase in the OS (Figure 1 a,b). Indocyanine green angiography (ICGA) with Heidelberg Retina Angiograph showed diffuse early and late hypercyanescence at the posterior pole in the OS (Figure 1 c,d). B-Scan Ultrasound using Nidek Co., Ltd. (Aichi, Japan) revealed diffuse retinochoroidal thickening with exudative retinal detachment in the OS (Figure 1 e,f). Spectral domain optical coherence tomography (SD-OCT) using RTVue XR Avanti (Optovue, Inc., Fremont, California, United States) showed diffuse choroidal thickening, loss of choroidal vascular architecture details with retinal pigment epithelium (RPE) undulations, sub-retinal fluid (SRF) with hyperreflective dots, and a fine epiretinal membrane in the OS (Figure 1 g).

After ruling out differential diagnoses, including sarcoidosis, tuberculosis, and syphilis, the patient was diagnosed with chronic SO based on the typical clinical presentation. The patient's treatment plan began with topical prednisolone acetate 1% hourly and topical homatropine 2% three times a day in the OS. However, the physician decided against administering intravenous methylprednisolone (IVMP) due to the presence of uncontrolled diabetes mellitus.

Therefore, as an alternative to IVMP, the patient was promptly started on immunosuppressive agents under the guidance and supervision of a rheumatologist. The treatment regimen included oral prednisolone 1 mg/kg daily (40 mg daily) and oral methotrexate 7.5 mg once a week.

**Figure 2:**
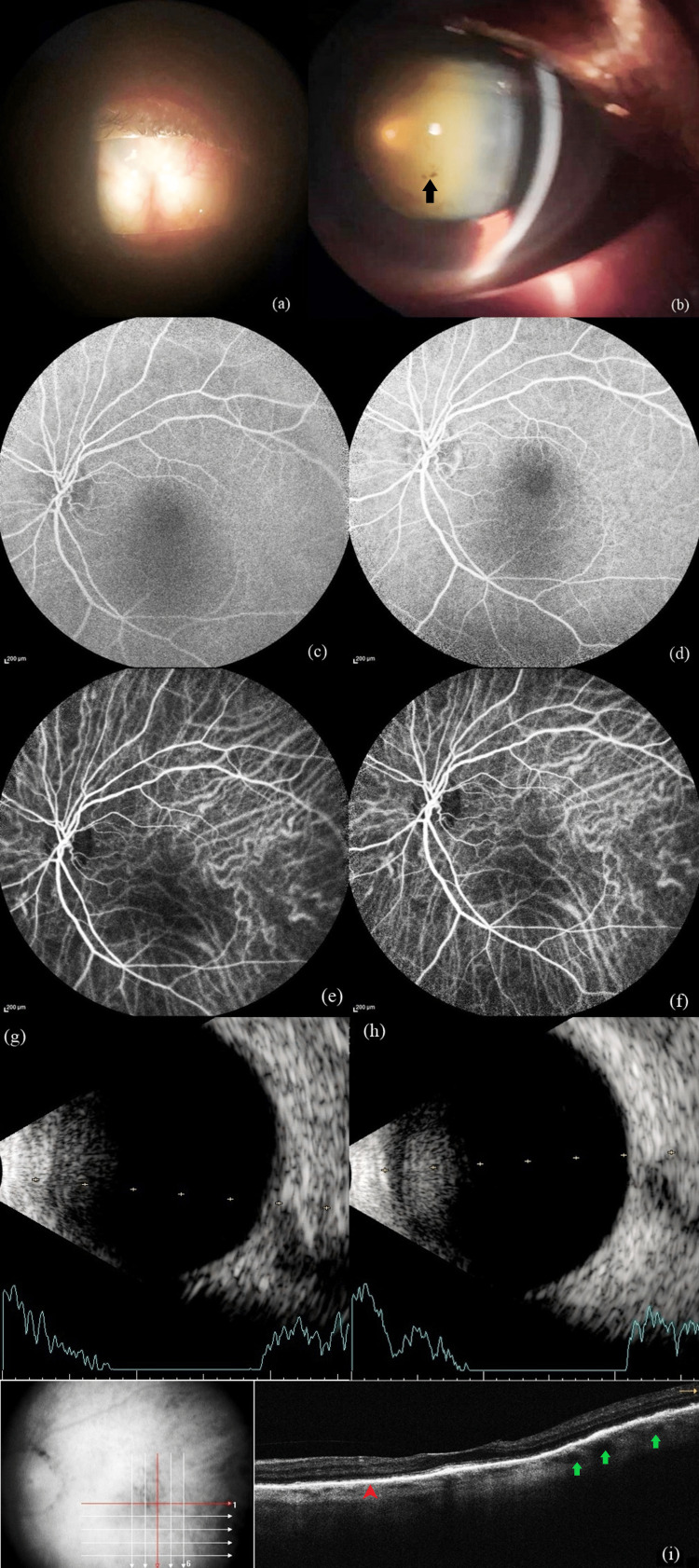
At the three-week follow-up visit after administering systemic immunosuppressive therapy. (a) Phthisical right eye. (b) Left eye showing pigments on the anterior lens capsule (black arrow) and a dense nuclear cataract. (c, d) Fluorescein angiography demonstrating the resolution of early and late-phase diffuse hyperfluorescence and significantly reduced disc staining. (e, f) Indocyanine green angiography showing normalization of early and late diffuse hypercyanescence noted at the first visit. (g, h) B-Scan ultrasonography displaying normal retinochoroidal thickness with resolution of exudative retinal detachment. (i) Spectral domain optical coherence tomography showing restoration of the normal choroidal vascular architecture (green arrows) along with resolution of retinal pigment epithelium undulations (red arrowhead) and subretinal fluid.

At the three-week follow-up visit, the BCVA had improved to 4/60 OS. Anterior segment imaging OU was performed during this visit as the patient showed symptomatic improvement (Figure 2 a,b). Mutton fat keratic precipitates, anterior chamber cells, and flare had resolved, but there was persistent pigment on the anterior lens capsule OS. The media remained hazy due to an advanced cataract OS (Figure 2 b). FFA revealed the resolution of diffuse early and late hyperfluorescence with a significant reduction in disc staining in the late-phase OS (Figure 2 c,d). ICGA demonstrated the normalization of diffuse early and late hypercyanescence OS (Figure 2 e,f). Ultrasonography (USG) B-Scan indicated the resolution of exudative retinal detachment and a reduction in retinochoroidal thickening OS (Figure 2 g,h). SD-OCT showed the resolution of RPE undulations and SRF, with a decrease in choroidal thickening and restoration of choroidal architecture details OS (Figure 2 i). Oral and topical steroids were tapered weekly, and systemic immunosuppression was continued with oral methotrexate. Unfortunately, the patient was eventually lost to follow-up.

## Discussion

SO has different clinical presentations in both acute and chronic stages, with no clear demarcation between the phases. The diagnosis is based on a past history of ocular trauma or surgical intervention and clinical signs showing granulomatous panuveitis. Multimodal imaging is crucial as it helps exclude a wide range of differential diagnoses, including causes of chronic granulomatous uveitis (sarcoidosis, tuberculosis, syphilis, bartonellosis, Lyme disease, and toxocariasis), exudative retinal detachment [Vogt-Koyanagi-Harada disease (VKH), central serous chorioretinopathy, posterior scleritis, uveal effusion syndrome, and masquerade syndromes (intraocular lymphoma)] [2]. Herein, we describe the utility of multimodal imaging in the timely diagnosis and management of chronic SO in a one-eyed patient.

Inflammation in SO primarily involves the choroid initially, presenting as posterior uveitis that ultimately evolves into panuveitis. Lesions are mainly situated in the choroid and demonstrate multiple foci of infiltrating lymphocytes on histopathology. Dalen-Fuchs nodules, seen clinically in acute SO as small, faint yellowish-white deep retinal/choroidal infiltrates, consist of epithelioid cells located between Bruch's membrane and the retinal pigment epithelium [3]. In the acute phase, enhanced depth imaging-OCT (EDI-OCT) shows choroidal thickening, RPE undulations, and loss of the physiologic choroidal vascular pattern. Additionally, there is the presence of subretinal fluid (SRF) and hyperreflective dots or fibrinous hyperreflective septae, which represent the shedding of photoreceptor outer segments. These alterations regress and completely resolve if prompt systemic therapy is initiated [4].

Chronic SO is characterized by sequelae such as chorioretinal damage, nummular scars, choroidal atrophy, subretinal fibrosis, and choroidal neovascularization (CNV) [5]. Multimodal imaging comes to the aid of the clinician, especially in delayed presentation when features of the acute phase have resolved, but sequelae of the chronic phase have not yet set in. B-scan ultrasonography is particularly useful where media haze due to advanced cataract, posterior synechiae, band-shaped keratopathy, and dense vitritis prevent a satisfactory view of the fundus. In SO, B-scan USG typically shows diffuse low to medium reflective choroidal thickening (in >60% of cases), and in some cases, serous retinal detachment at the posterior pole. B-scan helps differentiate between entities such as posterior scleritis and diffuse choroidal melanoma. On FFA, chronic SO shows hyperfluorescent window defects corresponding to areas with nummular scars and focal RPE and inner choroidal damage. Subretinal fibrosis and CNV reveal a gradual increase in hyperfluorescence and staining [6]. During the chronic stage of the disease, atrophic changes have been reported in the choroidal structure, regardless of the inflammatory status of the patient [7].

An important differential diagnosis of SO is VKH. These are clinically similar entities with similar signs on multimodal imaging, pathologies, major histocompatibility complex (MHC) haplotype, and histopathological findings. The most salient distinguishing feature between the above two entities is a past history of surgical intervention or ocular trauma in SO. Additionally, systemic signs such as headache, meningismus, tinnitus, vertigo, poliosis, vitiligo, and alopecia are usually seen in VKH [2].

ICGA is a crucial adjunct to FFA in the diagnosis of these entities, as the choroid is primarily involved in both pathologies. In acute SO, ICGA shows multiple hypocyanescent spots corresponding to choroidal infiltration with inflammatory cells and Dalen-Fuchs nodules on histopathological examination [8,9]. However, ICGA findings in chronic SO have not been frequently described in the literature, with inconsistent findings in rare case reports, such as one by Bernasconi et al. [8]. In their case series of a one-eyed 83-year-old male with chronic SO and a one-eyed 40-year-old male with acute SO, the predominant ICGA finding in both cases was hypofluorescent dark spots. The main pattern seen in their case of chronic SO was hypofluorescent areas in the intermediate and late phases of ICGA. This is in contrast to our reported case, which showed diffuse choroidal hypercyanescence in the intermediate and late phases. However, our findings on ICGA correspond to those described in chronic VKH. ICGA in chronic VKH reveals diffuse leakage in choroidal vessels ("fuzzy vessels") in the intermediate phase and diffuse choroidal hypercyanescence in the late phase [10]. The findings of chronic VKH can be extrapolated to those of chronic SO owing to the similarity in pathogenesis and histopathology [2,8]. Further studies to evaluate ICGA in chronic SO are warranted, with a larger sample size, to define pathognomonic imaging features in this rare entity.

## Conclusions

Our case of chronic SO, diagnosed with the help of multimodal imaging, showed features consistent with those described in the literature as noted above (including RPE undulations, SRF, obscured choroidal vascular architecture on OCT, early and late diffuse hyperfluorescence on FFA, and ICGA with prominent disc staining in the late phase). Marked resolution of these signs was noted in response to immunosuppressive therapy. Our report highlights the utility of multimodal imaging in this chronic condition with a myriad of equally puzzling differential diagnoses. Chronic SO may present with indistinct clinical signs, thereby posing a diagnostic dilemma. This may cause a delay in initiating treatment, which can prove to be highly detrimental, especially in one-eyed patients. Multimodal imaging is a critical tool in the timely diagnosis and accurate management of this sight-threatening condition.
